# Maternal positioning to correct occipito-posterior fetal position in labour: a randomised controlled trial

**DOI:** 10.1186/1471-2393-14-83

**Published:** 2014-02-24

**Authors:** Marie-Julia Guittier, Véronique Othenin-Girard, Olivier Irion, Michel Boulvain

**Affiliations:** 1 University of Applied Sciences Western Switzerland, 47 Avenue de Champel, Geneva 1206, Switzerland; 2 Department of Gynaecology and Obstetrics, Geneva University Hospitals and Faculty of Medicine, 47 Avenue de Champel, Geneva 1206, Switzerland

**Keywords:** Fetal head position, Occipito-posterior, Maternal position, Randomised controlled trial, Second stage of labour

## Abstract

**Background:**

The occipito-posterior (OP) fetal head position during the first stage of labour occurs in 10-34% of cephalic presentations. Most will spontaneous rotate in anterior position before delivery, but 5-8% of all births will persist in OP position for the third stage of labour. Previous observations have shown that this can lead to an increase of complications, such as an abnormally long labour, maternal and fetal exhaustion, instrumental delivery, severe perineal tears, and emergency caesarean section. Usual care in the case of diagnosis of OP position is an expectant management. However, maternal postural techniques have been reported to promote the anterior position of the fetal head for delivery. A Cochrane review reported that these maternal positions are well accepted by women and reduce back pain. However, the low sample size of included studies did not allow concluding on their efficacy on delivery outcomes, particularly those related to persistent OP position. Our objective is to evaluate the efficacy of maternal position in the management of OP position during the first stage of labour.

**Methods/design:**

A randomised clinical trial is ongoing in the maternity unit of the Geneva University Hospitals, Geneva, Switzerland. The unit is the largest in Switzerland with 4,000 births/year. The trial will involve 438 women with a fetus in OP position, confirmed by sonography, during the first stage of the labour. The main outcome measure is the position of the fetal head, diagnosed by ultrasound one hour after randomisation.

**Discussion:**

It is important to evaluate the efficacy of maternal position to correct fetal OP position during the first stage of the labour. Although these positions seem to be well accepted by women and appear easy to implement in the delivery room, the sample size of the last randomised clinical trial published in 2005 to evaluate this intervention had insufficient power to demonstrate clear evidence of effectiveness. If the technique demonstrates efficacy, it would reduce the physical and psychological consequences of complications at birth related to persistent OP position.

**Trial registration:**

ClinicalTrials.gov, http://www.clinicaltrials.gov: (no. NCT01291355).

## Background

**Figure 1:**
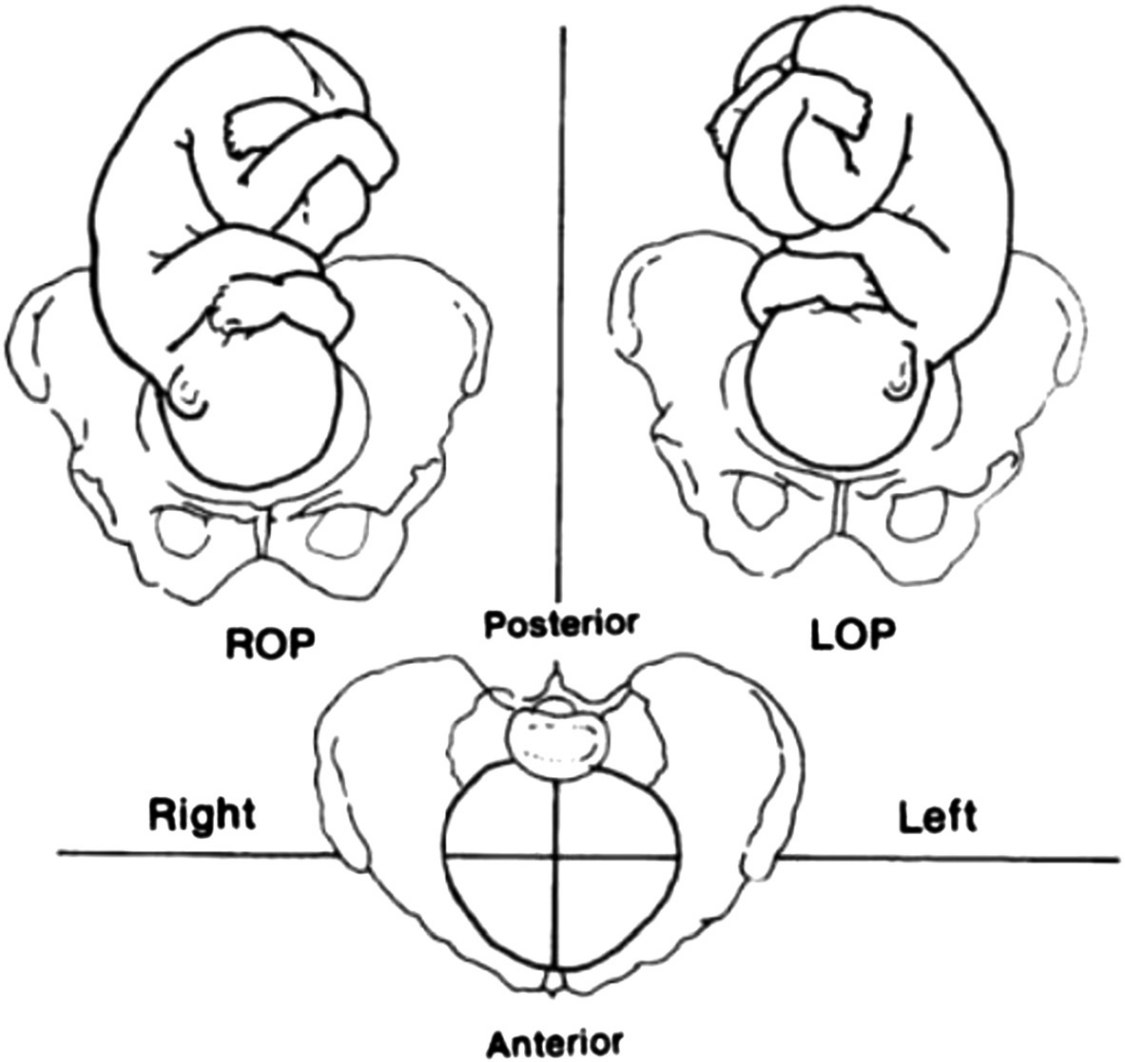
Fetal occipito-posterior (OP) position*. There are three OP positions: ROP (Right OP), LOP (Left OP), OS (occiput at sacrum). *This image is now in the public domain.

During the first stage of labour, 10% to 34% of fetuses are in occipito-posterior (OP) position (Figure 1) [1-3]. A cohort study of 1,562 nulliparous women reported an association between epidural analgesia and OP position [4], similar to a retrospective analysis of 30,839 deliveries conducted from 1976 to 2001 [5]. Parity, particularly nulliparity, appears to be also an aetiologic factor [2]. OP position is a malpresentation for delivery. Previous observations have shown an increase of short- and long-term complications, such as an abnormally prolonged labour, maternal and fetal exhaustion, instrumental delivery, emergency caesarean delivery, and severe perineal tears [5-8][9-12]. Similarly, in a prospective cohort study published in 2013, Carseldine et al. reported that OP position early in the second stage of labour is strongly associated with operative delivery: a total of 68% (13/19) women in the occipito-posterior group, and 27% (39/141) in the occipito-anterior group had an operative delivery (unadjusted: P < 0.001). Caesarean section was performed in 37% and 5%, respectively (P < 0.001) [13].

Usual care in the case of diagnosis of OP position is expectant management. Previous studies report that 72-90% of fetuses will spontaneously rotate to an anterior position during the first or second stage of labour [1,3]. Digital rotation of OP to anterior position has been described for the management during the second stage of labour. Although, it has the potential to successfully rotate the fetus and reduce the need for caesarean section, instrumental delivery, and other complications associated with OP position, it may also be traumatic for the fetal head and perineum.

Clinical diagnosis of the OP position is difficult as it is often associated with a deflection of the fetal head, and/or fetal head swelling, and oedema of the maternal cervix [14,15]. Several studies recommend verifying the clinical diagnosis of the fetal head position with ultrasound to increase the diagnosis of OP position early in labour [16,17]. According to Ramphul et al., an abdominal scan is easy to perform and is an accurate and acceptable method of diagnosing the fetal head position in the second stage of labour. It may also be useful in assessment prior to instrumental delivery [18].

### Literature review

A search of Medline and of the Cochrane Library was undertaken for relevant systematic reviews, meta-analyses, randomised controlled trials, and other clinical studies. A Cochrane review on the hands and knees’ posture in late pregnancy or labour for fetal malposition (lateral or posterior) concluded that the adoption of this posture 10 min daily in late pregnancy has the short-term potential to change the fetal position and reduce lumbar pain, but does not influence delivery outcomes [11]. The last randomised controlled trial on this topic was conducted by Stremler et al. in 2005 [19]. Similarly, the results reported a decrease of back pain associated with the hands and knees posture during the first stage of the labour, but the sample size of the study had insufficient power to demonstrate clear evidence to rotate OP in anterior position. The World Health Organisation encourages walking and changing of maternal position to promote spontaneous rotation of the fetal head in anterior position during the labour [20].

**Figure 2:**
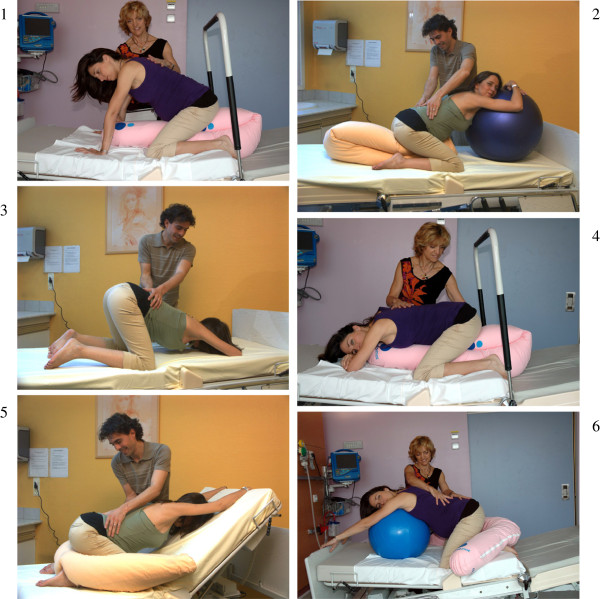
Six fitted hands and knees’ positions*. *Consent to publish these images was obtained by Dr Bernadette de Gasquet, author of the original book [21].

The book by de Gasquet described hands and knee positions to facilitate the rotation of the fetal head in anterior mode [21] (Figure 2). According to the author, these positions (resting on the knees, chest leaning forward and back stretched) would provoke fetal head OP rotation to anterior almost immediately. This technique seems simple to implement and acceptable to women according to previous studies that evaluated the experience of similar positions [11,22]. A relative disadvantage of this technique is the organisation of equipment for the mother (infusion, epidural, electronic blood pressure) and fetus (heart monitoring). Regarding the possible effect of the hands and knees’ position on the epidural anaesthesia, a French study conducted by anaesthetists showed that hyperflexion at the hips did not influence the expected epidural analgesia levels [23].

### Results of a pilot survey at the maternity of the Geneva University Hospitals

Epidemiological data on OP during labour are rare. Prior to starting the study and to elaborate the protocol, we conducted a retrospective analysis of 100 medical records randomly selected among all women who delivered during March 2010, to investigate the prevalence of OP position during labour and delivery, and mode of delivery. The data on the variety of presentation were absent for 25/100 medical records; 19/75 (25%) fetuses were diagnosed in OP position during the first stage of labour; and 9/75 (12%) had a persistent OP position for the second stage of the labour; of the latter, 7/9 were born by caesarean section. These results are more pessimistic than data reported in the literature. However, fetal outcome with OP is still poorly known and the contribution of this position to dystocia may be underestimated.

### Aims

The aim of our study is to evaluate the efficacy of hands and knees’ positions to correct fetal head position in OP during the first stage of the labour.

## Methods/design

We designed a randomised clinical trial, as it is the best study design to evaluate the effects of an intervention. We will compare the hands and knees’ positions (intervention) with expectant management (no intervention). “Expectant” is the standard care in our maternity in the case of OP diagnosis during the first stage of labour. There is no possibility of blinding, either the participant or the attending midwife or obstetrician, given the nature of the intervention. To introduce a “sham position” in the control group may be very difficult to imagine and interpreting the results would be cumbersome (is the proposed position beneficial or the control “sham” position increasing the risk or persistent OP?).

### Recruitment and intervention

Recruitment of women to the study will take place in the delivery room. During the period of recruitment study, we will perform systematically a transabdominal ultrasound to diagnose the fetal head position for each woman during the first stage of labour, to allow a reliable and early diagnosis of OP position.

#### Assessment of eligibility

Research midwives will verify eligibility for the study of all women presenting a fetus in OP position.

#### Inclusion criteria

The study will be limited to nulliparous and multiparous women during the first stage of labour with a cervical dilatation between 2 to 9 cm, a singleton pregnancy at term (≥ 37 weeks’ gestation), and an OP position diagnosed by ultrasound.

#### Exclusion criteria

Women under 18 years old, or who have a limited understanding of French, or who have attempted hands and knees’ positions previously during the first stage of labour.

#### Baseline

After consenting to participate in the study and prior to randomisation, women will be asked to complete a questionnaire including: sociodemographic data; resting position spontaneously adopted in late pregnancy; location of the pain perceived during uterine contractions (i.e., lower abdomen, back, other); perceived pain measured by the visual analogue scale (VAS) [18]; the comfort level of their position using a Likert scale (very comfortable, comfortable, neither comfortable nor uncomfortable, uncomfortable, very uncomfortable).

#### Randomisation

When women give their written consent to participate, a research midwife or the attending midwife will proceed with randomisation through a web-based system provided by the informatics department of the Geneva University Hospitals. Randomisation will be performed using randomly permuted blocks of varying size (4, 6 and 8), stratified by parity (nulliparous/multiparous) and epidural analgesia (yes/no). The ratio for hands and knees versus expectant management is 1:1. After confirming eligibility and consent, the system will return the allocation of the women to the midwife.

### Study participation

Table 1 summarises the chronology of the study conduct following the inclusion of participants.

**Table 1:** Summary of the chronology of study interventions

**Time 0:**	**Time 1:**	**Time 2:**	**Time 3**
**Diagnosis of occipito-posterior (OP) position of the fetal head**	**Randomisation and intervention**	**Evaluation of pain and comfort position 15 minutes after randomisation**	**Diagnosis of the fetal head position 1 hour after randomisation**
Action:	Action:	Action for both groups:	Action for both groups:
Confirmation of diagnosis by ultrasound (US).	“Control group” = expectative attitude for 1 hour in a comfortable position, excluding the 6 fitted hands and knees positions.	Women complete a short questionnaire about perceived pain (Visual Analogue Scale) and positional comfort (Likert scale).	Diagnosis of fetal head position by US.
			After the measure of the main outcome, the woman can freely take the position of her choice, including hands and knees.
Information and consent of the woman to participate to the study.			
	“Intervention group” = installation in one of the 6 fitted hands and knees’ position chosen by the woman for at least 10 minutes.		

### Interventions

#### Hands and knees position arm

Immediately after randomisation, women allocated in this group will be invited to choose one of the six positions described by Dr de Gasquet (Figure 2). According to this author, all these positions have the same impact on the OP position. The research midwife will present photographs of the six fitted positions (Figure 2) to help the women deciding which position is the best for her. These positions have three important points to be observed: 1) resting on the knees and, if necessary, on the hands; 2) the abdomen must be thrust forward; 3) the back is always stretched. A pillow should be placed between the legs of the woman in labour to limit discomfort. The woman decides if she wants to place her abdomen on a cushion or leave it unsupported. To help the women to take the appropriate position and to be sure that the position is correct, Dr de Gasquet has trained all midwives working in the delivery room of the maternity in the management of the OP position by specific positions of hands and knees. We shall recommend that participants to keep the position as long as they feel comfortable, but a minimum of 10 minutes is required. After this time, they can remain in the hands and knees’ position or change position if they prefer. Time spent in the evaluated position will be recorded in the data collection form.

#### Expectant management arm

Women allocated in this group will have the usual care in this obstetrical situation. Immediately after randomisation, they will stay in their position, other than the hands and knees’ position. After one hour and following ultrasound verification of the fetal head position, they will be given the option to adopt a hands and knees’ position, if they wish to do so. The position of the woman (standing, sitting, semi-sitting, lying on the back or the side) during this hour will be reported in the data collection form.

#### Both groups

Fifteen minutes after randomisation, women in both groups will complete a short questionnaire on two aspects previously measured just before randomisation, i.e., the perceived pain measured by the VAS and the comfort of their position evaluated by the Likert scale. One hour after randomisation, verification of the fetal head position will be performed, for assessing the primary outcome. Fetal head position will also be recorded at full dilatation of the cervix (before starting pushing efforts). The head position at delivery will also be reported in the data collection forms. Obstetrical and neonatal data will be collected in the medical record.

### Outcome measures

Our primary outcome measure will be fetal head in anterior position one hour after randomisation or at delivery if it happens first.

We have chosen one hour after randomisation rather than at full dilatation of the cervix as the time between randomisation and delivery can be very long. Thus, we estimate that it would be impossible and not ethic to control the position adopted by the participants for such a long time. In addition, one hour of expectant management is a duration that seems acceptable for both women and midwives in the control group, as they may be disappointed to be allocated to the control group. One hour after randomisation, women of both groups may adopt the position of their choice, hands and knees positions included.

Secondary outcomes: evaluation of the comfort of maternal positions; impact of the maternal position on the perceived pain; duration of the first and second stage of labour; mode of delivery according to the fetal head position; perineal status; neonate data (umbilical cord pH and apgar score < 7 at 5 minutes).

### Statistical analysis

Data analysis and reporting will be performed according to CONSORT guidelines for randomised controlled trials. A descriptive table of baselines characteristics will be reported for participants for both groups. Primary and secondary outcomes will be analysed on intention-to-treat basis. Sub-group analysis and eventually adjustment for the variables used for stratification (parity and epidural analgesia) will be performed. Means and their standard deviations will be calculated for continuous variables, and the statistical significance of differences between groups will be tested using Student’s *t*-test. Proportions will be compared between groups and differences will be tested using the chi-square test. If the distribution of the variables is not Gaussian, we shall use non-parametric statistical tests. The effect of the intervention will be estimated by the relative risk and its 95% confidence interval and p values to test the significance of the differences will be calculated.

### Sample size

We calculated that a sample size of 438 women (219 per group) will be needed to obtain a power of 80% with a two-tailed significance of 0.05 to show a statistically significant difference in the incidence of the main outcome measure. We hypothesized that the difference between groups in the proportion of fetuses rotating in anterior position one hour after randomisation will be 10% (10% in the control group versus 20% in the intervention group), a difference that we consider to be clinically significant.

### Feasibility

Approximately 15% of women will present a fetal OP position during the first stage of labour and this will concern 600 women per year at our maternity. By our previous experience, we estimate that around 50% of potentially eligible women will be screened (difficulty of the diagnosis during labour) and/or informed (depending on workload in the delivery rooms, emergencies). Thus, we estimate that study entry will be proposed to around 300 eligible women per year. We plan to enrol 150 women per year (12–13 per month). The required sample size could then be reached in around 35 months.

### Timetable

Total 42 months: commencing February 2010 with estimated completion June 2014.

1–2 months: regulatory approvals, preparation of the trial.

3–38 months: recruitment, intervention, data collection.

39–42 months: data analysis, reporting, peer review publications, conference presentations.

### Ethical aspects and safety considerations

The study protocol has been accepted by the institutional ethics committee of the Geneva University Hospitals (n° CER10-182). The safety of mothers and fetuses will be closely monitored as part of this study and a monitoring committee has been formed. Before enrolment, a research assistant or midwife will inform the women of the study. An information form will be available. A short period of reflection will be offered to decide upon participation due to an eventual emergency situation (imminent delivery). Women may withdraw consent at any time without negative consequences on the quality of care or staff attitude. Data will be treated on a confidential basis. Participants will be identified in the computerised database by a number that will be attributed at the same time as inclusion in the study. Study results will be reported in an anonymous form to protect the identity of participants.

## Discussion

### Potential and implementation of the findings

OP position increases the risks of maternal and fetal complications during labour and the delivery. However, the medical and midwife teams are currently powerless when faced with this diagnosis during the first stage of labour. Digital rotation of OP to anterior position has been described, but needs to be further evaluated for both efficacy and safety. Indeed, it may be traumatic for the maternal perineum and the fetal head. According to the literature, specific maternal positions, such as hands and knees, could facilitate the rotation of OP to anterior position. These postures appear to be easy to implement, safe for the mother and fetus, but their effectiveness must be evaluated. If hands and knees position is proven effective, it would be important to promote the diagnosis of OP position during the first stage of labour by ultrasound in order to act at that time as clinical diagnosis is difficult. If the fitted hands and knees’ position demonstrate proven efficacy, it would reduce maternal and fetal complications during labour and delivery, such as instrumented or caesarean delivery. Given the complications associated with persistent OP position, we consider that it is important to evaluate all interventions that may help fetuses to rotate in the anterior position.

## Competing interests

The authors do not report any potential competing interest.

## Authors’ contributions

Dr MJG and Pr MB had the original idea for the trial. Pr MB, Dr MJG, VO-G and Pr OI contributed to the trial. Dr MJG and Pr MB drafted the protocol, which was revised by all authors. Dr MJG and Pr MB are the guarantors. All authors read and approved the final manuscript.

## Pre-publication history

The pre-publication history for this paper can be accessed here:

http://www.biomedcentral.com/1471-2393/14/83/prepub
